# Choroidal thickness findings in two siblings with nanophthalmos by swept source-OCT: a case report

**DOI:** 10.1186/s13104-016-2359-x

**Published:** 2017-01-03

**Authors:** Hiroyuki Kaneko, Ari Shinojima, Mori Ryusaburo, Akiyuki Kawamura, Mitsuko Yuzawa

**Affiliations:** Division of Ophthalmology, Department of Visual Science, Nihon University School of Medicine, Nihon University Hospital, 1-6 Surugadai, Kanda, Chiyoda-ku, Tokyo 101-8309 Japan

**Keywords:** Axial length, Choroidal thickness, Hyperopia, Nanophthalmos, Optical coherence tomography

## Abstract

**Background:**

We investigated choroidal thicknesses at five sites in two siblings (four eyes) with nanophthalmos using swept-source optical coherence tomography.

**Case presentation:**

Case 1, a 51-year-old Japanese female with high hyperopia (Right: +20.5 Dioptors, Left: +19.5 Dioptors), had axial lengths of 15.6 mm in both eyes. Case 2, a 55-year-old Japanese male with high hyperopia (Right: +22.5 Dioptors, Left: +22.8 Dioptors), had axial lengths of 14.8 and 14.7 mm in the right and left eyes, respectively. Choroidal thickness was measured at five sites in each eye using swept-source optical coherence tomography; subfoveal, nasal, temporal, superior and inferior (the 4 non-subfoveal sites were measured 3000 µm from the fovea).

**Conclusion:**

The mean choroidal thickness was 355.8 ± 63.6 μm at the subfoveal, 466.3 ± 85.1 μm at the nasal, 274.8 ± 77.2 μm at the temporal, 396.8 ± 54.6 μm at the superior, and 480.8 ± 66.8 μm at the inferior (mean ± standard deviation) site. Choroidal thickness was maximal at the inferior site. The choroid was thinnest, in diminishing order, at the nasal, superior, subfoveal and temporal sites.

## Background

Nanophthalmos is a rare condition without other ocular or systemic anomalies, in which axial length (AL) is less than 20 mm and the eye volume is about two-thirds less than normal [1]. Nanophthalmos is associated with high hyperopia, narrow palpebral fissures, enophthalmos, shallow anterior chamber and scleral wall thickening. It can cause complications such as angle-closure glaucoma and uveal effusion [1]. Posterior segment findings in nanophthalmic cases reportedly include papillomacular folds, dilated vessels, cysts, pseudopapilledema, and thickened choroid [2, 3]. Genetic mutations are associated with nanophthalmos according to some reports, which described associations with the serine protease gene (PRSS56) and membrane frizzled-related protein gene (MFRP) [4, 5].

The choroid is the vascular layer containing pigmented tissues, and is located between the sclera and the retinal pigment epithelium (RPE). The choroid, which is essential for retinal function, provides oxygen and nourishment to the outer layer of the retina.

Recently, swept source-optical coherence tomography imaging (SS-OCT) has been used to evaluate choroidal changes. SS-OCT is known to have greater scanning speed and a longer wavelength (1050 nm), enabling deeper penetration through the RPE into the choroid, than other modalities. Hirata et al. reported choroidal thickness (CT) and volume at different sites in normal subjects using SS-OCT [6]. Demircan et al. detected subfoveal choroid thickening using enhanced depth imaging optical coherence tomography in patients with nanophthalmos [3]. There are many reports describing CT at the fovea itself, as well as at sites inferior, nasal, superior, and temporal to the fovea [6–8]. However, no studies have used SS-OCT to measure CT at different sites in nanophthalmos cases. The purposes of this study were to measure CT at different sites in two siblings with nanophthalmos and to compare the findings of our cases and those of normal eyes.

## Case presentation

Case 1: A 51-year-old Japanese female with nanophthalmic eyes. An objective refraction test (ORT) revealed high hyperopia in both eyes (Right: +20.5 Diopters (D), Left: +19.5 D). AL was 15.6 mm in both eyes.

Case 2: A 55-year-old Japanese male, the brother of Case 1, with nanophthalmic eyes. ORT revealed high hyperopia in both eyes (Right: +22.5 D, Left: +22.8 D). AL was 14.8 mm in the right and 14.7 mm in the left eye.

**Fig. 1:**
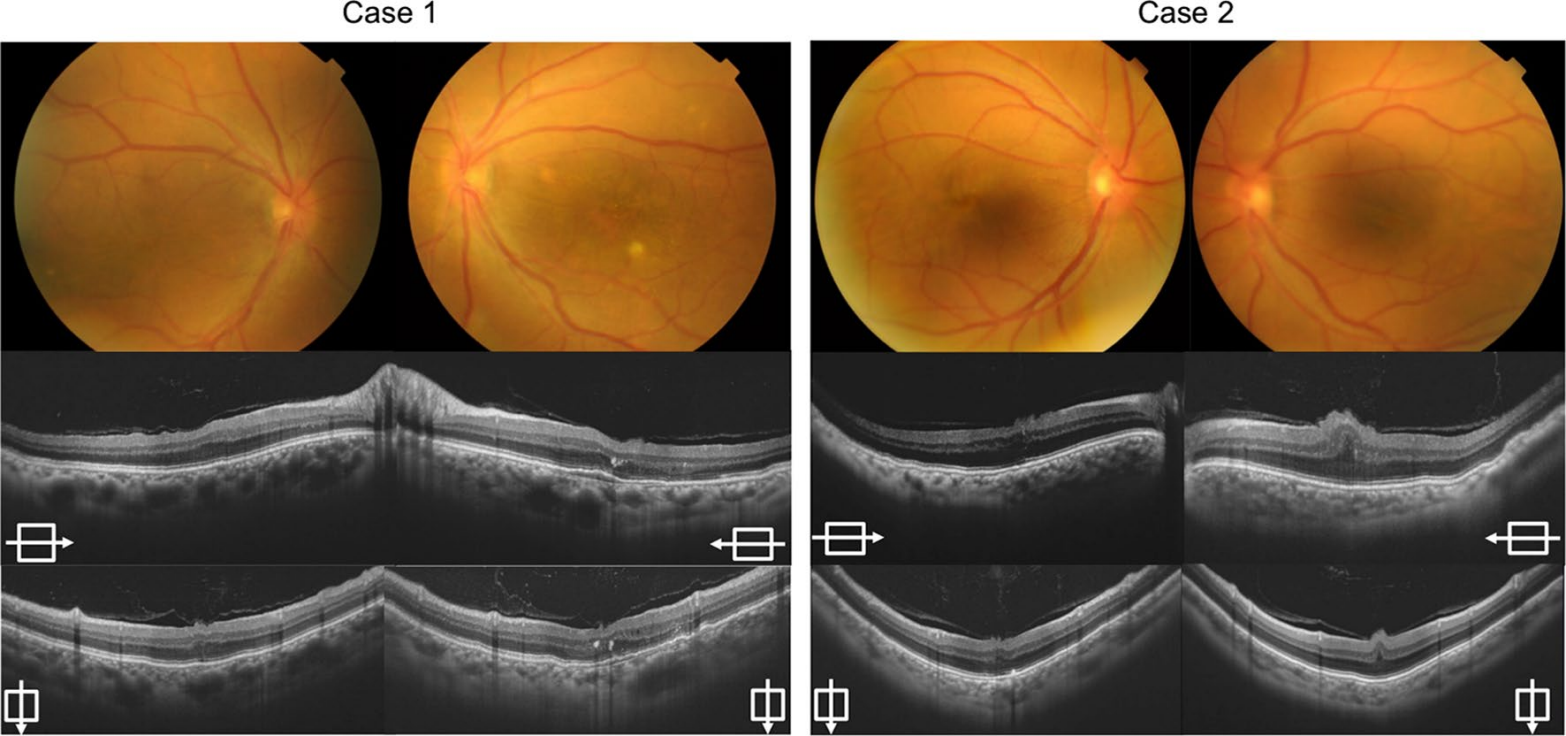
The *color* fundus photographs and swept-source optical coherence tomography images of both cases. The color fundus photographs of Case 1 and Case 2 show macular folds and dilated vessels (*top*). The swept source-optical coherence tomography images reveal macular folds and thickened choroid at the subfoveal point in all four eyes. The *horizontal arrows* indicate *horizontal images* through the fovea (*middle*). The *vertical arrows* indicate *vertical images* through the fovea (*bottom*)

The AL was measured using an intraocular lens master (Carl Zeiss, Germany). Retinal folds at the macula and dilated veins were detected in all 4 eyes. SS-OCT revealed retinal folds at the fovea and a thickened choroid in all 4 eyes (Fig. 1).

**Fig. 2:**
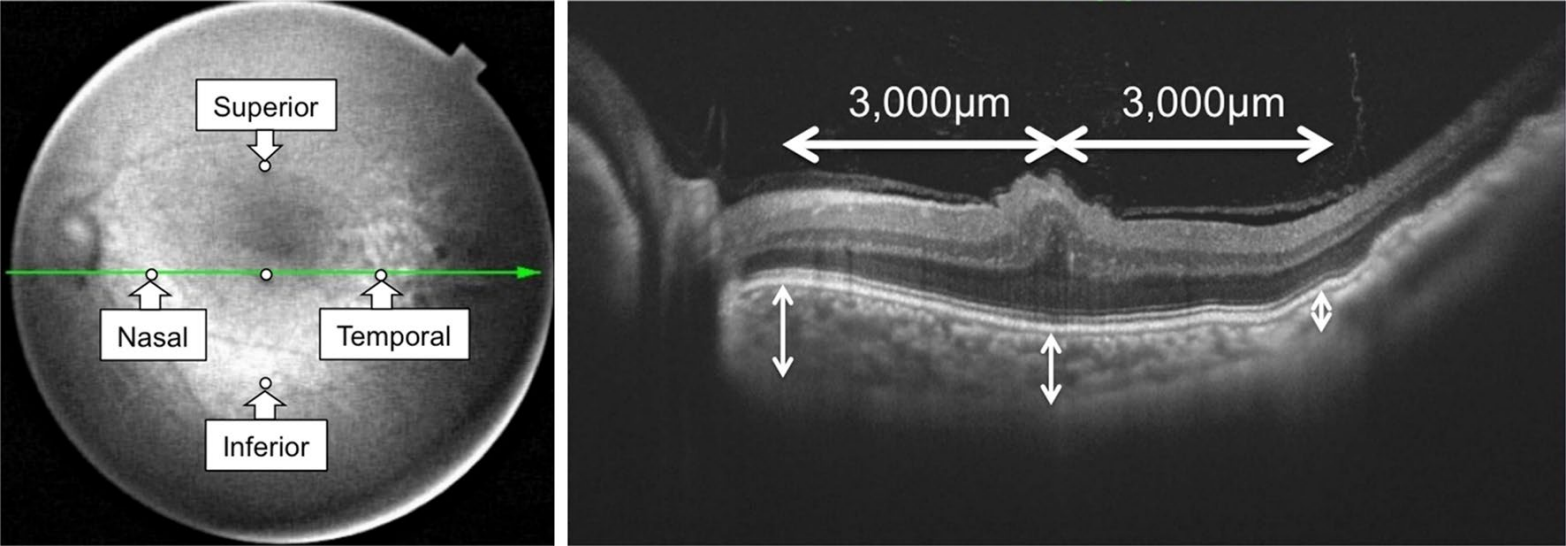
The infrared image (*left*) and the swept source-optical coherence tomography image (*right*) from Case 2 (*left*). The *horizontal line* is through the fovea on the infrared image. *White circles* indicate the fovea and the other four sites (nasal, temporal, superior and inferior, each measured 3000 µm from the fovea). (*Right*) Choroidal thickness was measured from the *bottom* of the hyper-reflective line of the retinal pigment epithelium to the chorioscleral border at each measurement point

CT was measured manually at the fovea and 4 other sites (nasal, temporal, superior and inferior to the subfoveal measurement point) 3000 µm from the foveal site in each eye using SS-OCT (DRI OCT-1 “Atlantis”; Topcon Medical Inc., Tokyo, Japan) (Fig. 2). CT was measured manually with a built-in caliper (H.K.), according to Rhaman et al. [8]. CT was measured from the bottom of the hyper-reflective line of the RPE to the chorioscleral border at each measurement point. The mean CT values at each site in the four eyes were analyzed.

## Conclusions

The mean CT measurements are shown in Table 1. CT was maximal at the inferior site. The second largest CT value was measured at the nasal site, followed in order by the superior, subfoveal and temporal sites, with the latter being the thinnest portion of the choroid.

**Table 1:** Average choroidal thickness at five sites

Site	Choroidal thickness (mean ± standard deviation) µm
Inferior	480.8 ± 66.8
Nasal	466.3 ± 85.1
Superior	396.8 ± 54.6
Subfoveal	355.8 ± 63.6
Temporal	274.8 ± 77.2

There are reports describing CT at the nasal site as being less than that at the other 4 sites (subfoveal, temporal, inferior and superior) in healthy subjects [6, 7]. In healthy Japanese subjects, the mean CT at the fovea was reported to be 202.6 ± 83.5 µm, the mean CT at the nasal measurement point 152.5 ± 71.1 µm [7]. In our cases, however, the mean CT at the inferior and nasal sites were greater than those at the same sites in healthy subjects. The CT values at the subfoveal and nasal sites were also greater than those at the same sites in healthy Japanese subjects, serving as controls.

The ALs in the four eyes of our two subjects were equivalent to those seen in nanophthalmos cases examined between the postnatal period and 2 years of age [9]. Furthermore, in our cases, ultrasonic B-mode imaging revealed thickened sclera and short AL. Therefore, it was suspected that development of the sclera in these four eyes had stopped during this early period of life. It has been hypothesized that retinal folds are molded by normal retinal development with arrested scleral development [10]. This hypothesis may apply to our cases.

Primordial tissues of the choroid and sclera are comprised of mesenchymal cells around an optic cup. From the 6-week embryonic stage, differentiation into choroid and sclera starts via induction of the RPE. Therefore, we theorized that development of the choroid had stopped in these siblings with nanophthalmos, since the primordial tissues of the choroid and sclera were the same. If the eyeball develops normally, the choroid may extend from the disc in the temporal direction, and the nasal site is thinner in normal eyes. In these four eyes, however, the nasal site near the disc was thickened due to poor extension of the eyeball.

It is easy to obtain non-invasive OCT images, even in relatively young patients. Salchow et al. reported only 4 (3.3%) of 121 subjects younger than 18 years to be uncooperative [11]. Based on only one horizontal OCT image, we can surmise that strong hyperopia is present with a short AL, if the OCT image has findings indicating that the nasal choroid is thicker than the temporal choroid, there are papillomacular folds and non-inflammatory cells are present in the vitreous cavity.

The major limitation of this study is the small number of patients. Also, we were not able to perform a genetic analysis in these cases. Further study is needed to clarify the features of and genetic contributions to nanophthalmos.

In conclusion, CT was maximal at the inferior site. The choroid was thinnest, in diminishing order of CT measurement values, at the nasal, superior, subfoveal and temporal sites. Unlike normal eyes, the nasal site of the choroid in these four nanophthalmic eyes was thickened, presumably due to arrest of choroidal and scleral development.

## Abbreviations

AL: axial length
CT: choroidal thickness
D: diopters
ORT: objective refraction test
SS-OCT: swept source-optical coherence tomography
RPE: retinal pigment epithelium
